# Post-Traumatic Stress Disorder among Cardiac Patients: Prevalence, Risk Factors, and Considerations for Assessment and Treatment

**DOI:** 10.3390/bs5010027

**Published:** 2014-12-23

**Authors:** Heather Tulloch, Paul S. Greenman, Vanessa Tassé

**Affiliations:** 1 Division of Prevention and Rehabilitation, University of Ottawa Heart Institute, Ottawa, Ontario K1Y 4W7, Canada; 2 Department of Psychology, Université du Québec en Outaouais, Gatineau, Quebec, J8X 3X7, Canada; E-Mails: Paul.Greenman@uqo.ca (P.G.); tasv01@uqo.ca (V.T.); 3 Montfort Hospital Research Institute, Ottawa, Ontario, K1K 0T2, Canada

**Keywords:** posttraumatic stress, cardiovascular, assessment, treatment

## Abstract

There is increasing awareness of the impact of post-traumatic stress disorder (PTSD) on physical health, particularly cardiovascular disease. We review the literature on the role of trauma in the development of cardiovascular risk factors and disease, aftermath of a cardiac event, and risk for recurrence in cardiac patients. We explore possible mechanisms to explain these relationships, as well as appropriate assessment and treatment strategies for this population. Our main conclusion is that screening and referral for appropriate treatments are important given the high prevalence rates of PTSD in cardiac populations and the associated impact on morbidity and mortality.

## 1. Introduction

Post-traumatic stress disorder (PTSD) is a common psychiatric disorder that can affect individuals who experience or witness a life-threatening or violent event. Individuals with PTSD experience a number of distressing symptoms that fall into three main categories: (1) re-experiencing symptoms such as intrusive thoughts, nightmares or flashbacks; (2) avoiding stimuli or reminders of the event; and (3) physiological arousal (e.g., hypervigilence, exaggerated startle response). The estimated lifetime prevalence rate is 4.4% for developed nations [1], however, the Diagnostic and Statistical Manual of Mental Disorders-5th edition (DSM-5, 2013) reported lifetime prevalence for the United States (US) to be 8.7%.

Understandably, research on PTSD has focused on psychiatric symptoms, with less emphasis on the relationship between PTSD and physical functioning. However, there is increasing awareness of the impact of PTSD on physical health, in particular, cardiovascular disease (CVD). In fact, a US national epidemiologic survey with almost 35,000 participants revealed that respondents with PTSD were more likely than non-traumatized respondents to report diagnoses of diabetes mellitus, liver disease, stomach ulcer, gastritis, HIV, arthritis, angina pectoris, tachycardia, hypercholesterolemia, and other heart diseases [2]. The risk of developing cardiovascular risk factors (e.g., hypertension, cardiac hyper reactivity) and coronary artery disease in patients with PTSD has also been established of late [3,4,5]. For example, a recent meta-analysis [4] showed that PTSD is associated with a 55% increase in risk for heart disease or cardiac-specific mortality; this relationship remained significant after adjustment for numerous clinical, demographic, and psychosocial factors, including depression.

As survival rates from myocardial infarction (MI) and cardiac arrest (CA) have increased with advanced medical technology both in the community (e.g., automated external defibrillator) and in-hospital [6,7], more people are living to remember these life-threatening events. As a result, the relationship between cardiac events and subsequent traumatic symptoms is also coming into light [8]. These patients may be prone to develop re-experiencing (e.g., recalling the cardiac event or defibrillator shocks, dreams of cardiac arrest, flashbacks of medical interventions and surgical procedures), avoidance (e.g., avoid reminders of the cardiac event such as the location of the event, the hospital, medication, situations in which heart rate increases such as exercise or sexual activity), and arousal symptoms (e.g., preoccupation with heart rate or chest pain; insomnia).

Prevalence rates of PTSD vary across cardiac populations: 4%–24% of patients who experience acute coronary syndrome [8,9,10,11,12], approximately 20% of patients with implantable cardioverter defibrillators (ICDs; [13]), and 19%–38% of those who suffer a cardiac arrest reported clinically significant symptoms of post-traumatic stress [8,13,14]. Overall, prevalence rates tend to attenuate somewhat with time. For example, 24% of MI patients in one investigation met criteria for PTSD in the first month post-MI and, albeit double the national average, only 15% continued to report these symptoms at 9 months [12]. Unfortunately, the risk of recurrent acute coronary syndrome (*i.e.*, heart attack or angina) is double for patients who develop PTSD, as compared to those without this diagnosis [9].

Clearly, the relationship between trauma and cardiovascular disease is complex, both in the development of CVD and its management. The remainder of this paper consists of a review of the literature concerning the role of trauma in the development of cardiovascular risk factors and disease, aftermath of a cardiac event, risk for recurrence in cardiac patients, possible mechanisms explaining these relationships, and appropriate assessment and treatment strategies for this population.

## 2. Trauma and PTSD Increase the Risk of CVD Development

As noted above, a history of PTSD has been associated with risk factors for CVD including hypertension, dyslipidemia, obesity, and diabetes in veterans and endothelial dysfunction and systemic inflammation civilian populations [15,16,17]. Coronary artery calcium (related to atherosclerosis), in veterans without known coronary artery disease, is also more prevalent among those with PTSD as compared to non-PTSD patients across all cardiovascular risk categories as measured by the Framingham risk score [18]. Findings from these and other cross-sectional studies have now been replicated in prospective studies [19,20,21], allowing for more causal inferences. For example, data from the World Trade Center Registry (N = 39,324) revealed that adults exposed to the 9/11 terrorist attack who developed PTSD had increased risk for heart disease 3 years, on average, after the disaster (HR = 1.68; 95% CI 1.33-2.1; [20]). Even more compelling was a prospective study that followed Vietnam-era veteran twins (N = 281 pairs) for a median of 13 years [22]. The incidence of heart disease was more than double in twins with (22.6%) than those without PTSD (8.9%). Results remained robust even when analyses were adjusted for lifestyle factors, other CVD risk factors, and major depression. Objective measures (*i.e.*, cardiac positron emission tomography) provided additional proof of the reduced cardiac function in the PTSD *vs.* non-PTSD cohort [22]. Finally, a meta-analysis of six prospective studies investigating the relationship between PTSD and cardiac disease, including patients free of CVD at baseline, concluded that PTSD is independently associated with increased risk for incident CVD without (HR = 1.55; 95% CI 1.34-1.79) and with adjustment for depression (HR = 1.27; 95% CI 1.08-1.49; [4]). Additional studies supporting the link between PTSD and CVD continue to be published [23,24].

In addition, a dose-response relationship appears to exist such that those with higher levels of distress are at considerably greater risk of cardiotoxic effects [21,25]. For example, one prospective study with 1059 women revealed that those with 5 or more symptoms of PTSD were at 3.21 times the risk of incident coronary heart disease compared to those with no symptoms [26]. Similar results were found in 15-year follow-up study of veterans with no history of heart disease at baseline: A 5-point increase in trauma symptoms was associated with a 20% increase in mortality risk (Boscarino, 2008) [19]. Similarly, Kubzansky and colleagues [25] reported that for each standard deviation increase on the *Mississippi Scale for Combat-Related PTSD*, the age-adjusted relative risk for all CVD outcomes (e.g., MI, angina) in a cohort of men who served in the military was 1.2 (95 CI 1.05-1.51). Finally, a large population sample that included over 3000 US adults (2% diagnosed with PTSD and 53% had trauma symptoms but not PTSD) revealed that those with trauma experiences were at increased risk of angina and heart failure; the PTSD group’s risk doubled (angina; OR = 2.4) and tripled (heart failure; OR = 3.4) that of the non-traumatized group [27].

## 3. PTSD in the Aftermath of a Cardiac Event

Anxiety is a normal response following a major cardiac event (e.g., heart attack, bypass surgery). However, in its extreme form of PTSD, significant distress, poor function, and detrimental outcomes may occur. As outlined above, the rates of PTSD across cardiac populations vary, but clearly surpass those of the general population. Although studied to a lesser degree than PTSD related to the development of CVD, it is equally important to understand what factors predict a PTSD diagnosis after a cardiac event, as well as the potential for increased cardiac morbidity and mortality in these patients.

Previous research has reported numerous predictors of PTSD after a cardiac event including: (1) cardiac-event factors including perception of threat to life, fear at the time of the event, severity of chest pain, illness comprehension, prior MI or cardiac hospitalization; (2) dissociation at the time of hospital admission and intensity of acute stress disorder; (3) psychiatric history such as depressive symptoms and prior traumatization; (4) personality type including alexithymia, repressive coping style, and type D personality; (5) sociodemographic factors (e.g., age, female); and (6) lack of social support [9,10,11,12,28,29]. Further, patients who experience cardiac arrest develop PTSD at twice the rate of those who experience MI [8]. Specific to ICD patients, risk factors for psychological distress include younger age (>50 years), being female, premorbid psychiatric diagnoses, poor social support and receiving multiple shocks (>5; [30,31]).

Previous trauma as a risk factor is not surprising. A recent study that investigated lifetime trauma exposure among 1021 cardiac patients found that 99% had been exposed to at least one traumatic event. In fact, the average number of traumatic events experienced by these patients was 5.6 [32]. These researchers reported a 38% greater risk of adverse cardiovascular outcomes (*i.e.*, CVD events and all-cause mortality) among participants in the highest exposure quartile (adjusted for age, sex, race, income, education, depression, PTSD, GAD, smoking, physical inactivity and illicit drug use; [32]).

Few studies have investigated PTSD post-cardiac event prospectively. One study recruited 73 patients 6 months post-MI and followed them for one year found that those with elevated PTSD scores were more likely to be readmitted to hospital for cardiac symptoms [33]. When studied in a larger sample (*n* = 297), similar findings were found [34]. Specifically, a 10-point increase in the PTSD score predicted a 42% increased risk of non-fatal or major CVD-related hospital admission during the follow-up period (2.8 years on average). Edmondson and colleagues [35] took the measurements one step further and assessed whether acute coronary syndrome-induced PTSD symptoms increase risk for major adverse cardiac events (*i.e.*, unstable angina, MI or emergency revascularization) and all-cause mortality in 247 patients. Results showed a strong relationship in the unadjusted analyses (HR = 2.42; CI, 1.47-6.12); however, when adjustments were made for demographic, medical and depression covariates, the strength fell below significance (HR = 1.36; CI, 0.54-3.46). Yet when data from the above studies were combined in a meta-analysis [9], risk for recurrent cardiac events and mortality for patients with post-traumatic stress symptoms doubled in the first to second year after their acute coronary event. Risk appears to be worse still for those with implanted cardiodefibrillators; patients with PTSD were 3.2 times more likely to die within 5 years as compared to those with none to moderate symptoms of trauma [13]. To truly understand the risk of recurrence, additional studies with larger samples and diagnostic interviews to confirm self-reported trauma symptoms are needed.

## 4. Biological and Behavioural Mechanisms

A number of hypotheses regarding potential biological and behavioural pathways by which PTSD is related to CVD have been put forth. Various physiological markers, most commonly blood pressure, heart rate, and electrodermal activity have been studied in patients with PTSD, and suggest involvement of the sympathetic nervous system. When a person is faced with challenges such as infection or danger, the sympathetic nervous system and HPA axis are activated. Typically, these systems turn off when the threat is removed. This bodily fluctuation in response to these external elements is called allostatis. Extended or chronic stress leads to increased neural or neuroendocrine responses, leading to “allostactic load” and adverse effects on the body [36]. Ultimately, this wear and tear and system over activation may lead to atherosclerosis and cardiovascular system damage.

Neurobiological abnormalities associated with PTSD such as deregulation of the hypothalamic-pituitary-adrenal (HPA) axis may increase vulnerability to chronic diseases by weakening the immune system. PTSD is related to cardiovascular reactivity including chronically elevated pro-inflammatory activity and endothelial dysfunction, all of which promote the development and exacerbation of heart disease [37]. Research has shown that people exposed to two or more childhood traumas have elevated C-reactive protein (an inflammatory marker) in adulthood [38,39,40]. Similar results have been observed in trauma occurring in adulthood, albeit with small sample sizes [41,42]. In a large-scale prospective study with stable CVD patients, O’Donovan and colleagues [43] reported that higher lifetime trauma exposure was associated with elevated inflammation (as measured by interleukin-6 (IL-6), tumor necrosis factor-α (TNF-α), C-reactive protein and resistin, which is related to an accelerated rate of CVD progression.

Another cardiovascular risk factor—hypertension—has been associated with hyperarousal, a diagnostic symptom and hallmark of PTSD. In fact, systolic and diastolic blood pressure values are approximately 10mmHg higher [5,44]. One study reported [5] that 34% of their sample of young veterans had hypertension, a rate much higher than the general population in this age category (11%). Finally, exaggerated threat perception has been observed in trauma survivors [44,45], and perceived threat elicits the biological stress responses as discussed above. As such, these patients experienced prolonged system activation that puts them at risk of further CVD development or exacerbation.

Unhealthy behaviours are also associated with PTSD and are risk factors for cardiovascular disease, including poor diet and alcohol use, sedentary behaviour and smoking. Data from over 1000 patients in the Heart and Soul Study [46] revealed that those with PTSD had higher rates of physical inactivity and greater smoking history (*i.e.*, more pack years). Henrickson and colleagues [32] observed the same physical inactivity and smoking tendency, while others have shown that PTSD leads to alcohol and illicit drug use in patients with cardiac disease [47].

Medication non-adherence is especially problematic for those with PTSD, and may contribute to increased CVD risk [46,48]. For example, in a sample of 724 veterans affairs patients, Kronish *et al.* [48] found that those with PTSD (35%) were less likely to take their medication as prescribed (12% *vs.* 9%), and were more likely to forget to take medications (41% *vs.* 29%) and skip dosages (24% *vs.* 13%). These associations remained significant after adjusting for demographic factors, medical comorbidities, social support, depression, and alcohol use (OR = 1.47; 95% CI 1.03-2.10 for not taking as prescribed and OR = 1.95; 95% CI 1.31-2.91 for skipping medications).

Another possible explanation of worse cardiac outcomes in patients with PTSD may be related to their tendency to delay presentation to hospital with acute coronary symptoms [49]. In fact, Newman and colleagues [49] reported that of 241 patients with acute coronary syndrome, those with PTSD (18%) took 15 hours, on average, longer than the non-PTSD cohort to present to hospital. After adjustment for other demographic and clinical variables, the mean pre-hospital delay was 173% longer for patients with *versus* without PTSD symptoms. This value is staggering considering that even a 30-minute delay in treatment for acute coronary syndrome is associated with greater morbidity and mortalityrates [50]. 

It may be that the symptom overlap between these two conditions (*i.e.*, tachycardia, dyspnea, diaphoresis) is confusing to patients with PTSD, leading them to discount the possibility of cardiac problems and subsequently delay medical evaluation. It may also be that the trauma history contributed to fear and perceived helplessness regarding the event [51], and that these emotions prevented them from accessing care.

## 5. Assessment Considerations

The results of the above studies underscore the importance of screening for trauma symptoms in cardiac patients, however, the question remains as to which assessment measure is most suitable. The choice of instrument is important as it may influence prevalence rates and treatment decisions. If not accurately assessed, treatment planning will be misled. To date, rates of PTSD in cardiac patients vary widely, likely due in part to the differences in measurement and patient population (e.g., MI, cardiac arrest, ICD; [8,52]).

A number of assessment measures are available and have been employed with cardiac populations. Self-report questionnaires are often used as they are easy to administer and score, and they save valuable time for researchers and clinicians. Popular measures include: (1) *PTSD-checklist* (PCL), a validated 17-item event-specific scale that inquires about symptoms in the last 30 days [53]; (2) The *Impact of Events Scale—Revised* (IES-R; [54]) a 22-item self-report measure with three subscales—intrusion, avoidance and hyperarousal; (3) *Posttraumatic Diagnostic Scale* (PDS; [55]), a 48-item self-report measure with 4 sections that assess all diagnostic criteria for PTSD in DSM-IV; and (4) the *Post-traumatic Symptoms Scale* (PTSS-10; [56]), a brief 10-item questionnaire assessing PTSD symptoms. Another ICD-specific questionnaire, The *Florida Shock Anxiety Scale* [57], evaluates patients’ anxiety about their ICD and its functioning.

Alternatively, structured clinical interviews based on the DSM may be conducted by clinicians or trained research personnel to assess for PTSD symptoms. These measures typically evaluate patients’ PTSD symptoms on 4 categories: (i) experience of a traumatic life-threatening event; (ii) persistent re-experiencing of the event; (iii) persistent avoidance of associated stimuli; and (iv) persistent symptoms of increased arousal regarding traumatic events. Previous research with cardiac patients has included the following: (1) *Structured Clinical Interview for the Diagnostic and Statistical Manual* [58], the most widely used criterion against which other measures have been validated; (2) *Computerized Diagnostic Interview Schedule* (CDIS; [59]), a validated computer-based standardized psychiatric interview for DSM-IV administered by trained research personnel; and (3) The *World Health Organization Composite International Diagnostic Interview* (CIDI; [60]). The *Mini International Neuropsychiatric Interview* (MINI [61]) is another diagnostic interview that has been used to assess other psychiatric disorders in cardiac patients, and could also be considered for evaluation of PTSD. It is important to note that the criteria for PSTD have been recently revised in the DSM-V; however, to our knowledge, no published structured clinical interviews are available to date.

Although they are more time-consuming, results from three studies [8,28,52] suggest that the “gold standard” for identifying PTSD in cardiac patients remains the diagnostic interview. To illustrate, Einsle and colleagues [52] found inflated prevalence rates when questionnaires were used (*i.e.*, IES-R and PTSS-10) *versus* the SCID diagnostic interview. Specifically, diagnostic criteria for PTSD were met by 29% of patients via the PTSS-10, 7.6% with the IES-R, and 4.8% with the SCID. Although O’Reilly and colleagues [8] found similar prevalence rates of PTSD when using the SCID diagnostic interview and the PDS self-report questionnaire, relatively poor agreement between the measures was evident (e.g., only 3 individuals were identified in common). Others have noted that self-report questionnaires do not discriminate well between negative emotions and do not always map well onto diagnostic categories, which limits their usefulness [24]. We argue that a screening questionnaire such as those above or others designed for general medical settings (e.g., *Primary Care PTSD Screen*; [62]) may be used initially and, if scores surpass clinical cut offs, a full PTSD assessment ought to be carried out by a qualified clinician. We also advocate for the integration of trauma assessment into the overall cardiac health assessment conducted by cardiologists and other health professionals, as doing so may improve detection and lead to essential treatment for trauma symptoms.

## 6. Treatment Considerations

The initiation of psychological treatment of the cardiac patient with PTSD can occur in the primary care or cardiac-specific setting. Unfortunately, medical clinics function under incredible time constraints and typically lack in-house mental health professionals to whom they may refer. Along with others [63] we advocate for more cardiac psychologists in health care facilities, but until that time, we suggest that physicians, cardiologists, and other health care providers create a referral list of mental health care providers in the community who have experience providing these specialized services.

In general, it is important to reassure cardiac patients that their symptoms are normal and common reactions to a life-threatening event. Providing psychoeducation regarding PTSD and other psychological disorders related to cardiac disease is an important first step to help normalize symptoms, and to avoid exacerbation of symptoms due to avoidance. Initial interventions would include sleep hygiene, behavioral sleep strategies, and relaxation techniques such as diaphragmatic breathing, meditation/mindfulness, and progressive muscle relaxation to help regulate high arousal and curb the often-reported sleep disturbances.

Evidence-based psychological treatments for PTSD such as cognitive-behavioural therapy (CBT) are likely appropriate for PTSD in cardiac patients [64,65,66]. However, only two cardiac-specific PTSD treatments studies have been published to date. The first pilot study (N = 14) offered 4–5 sessions of trauma-focused cognitive-behavioural therapy to MI patients with PTSD. Results showed improved PTSD and depression scores and better risk-factor control among patients in the intervention [33]. These researchers followed up with a larger prospective randomized controlled trial (N = 65) that assigned ACS patients to 3–5 imaginal exposure therapy sessions or 1–3 education sessions only [67]. Although the study was not powered to detect group differences, reductions in PTSD symptoms were reported, especially for patients with high baseline PTSD symptoms. Importantly, the safety of engaging in such treatment was proven.

In addition to CBT, which has garnered substantial empirical support for the treatment of PTSD in the general population, it is also important to consider other psychotherapeutic approaches that have shown promise and that target known risk and protective factors for PTSD. For example, the lack of social support has been long been identified as a principal risk factor for PTSD in the general population without cardiac disease, even when controlling for personality traits and the intensity of the traumatic event or events to which people might have been exposed [68]. Conversely, a large body of research suggests that the perception of the emotional availability and presence of significant others (e.g., friends, colleagues, romantic partners) acts as a buffer against the development of PTSD and can play a crucial role in recovery from it [69,70,71]. Stated simply, people who feel isolated and alone are more likely to develop PTSD than are people with close connections to others, and they have more difficulty working through the symptoms of PTSD than do people who perceive high levels of social support in their lives.

Recent research reaffirms these findings from the general population with cardiac patients. For example, perceived social isolation has been identified as an important factor predicting PSTD symptoms in acute coronary syndrome patients [10]. Nachar and colleagues [72] found that participants’ heart rate reactivity increased during a trauma-related discussion with their significant in the laboratory if they perceived their partner’s support to be negative (*i.e.*, if they felt misunderstood or unheard). Another study found that partners were even more susceptible to PTSD than were cardiac patients themselves [73]. These findings are not surprising given the established link between social support and recovery from heart disease [74]. In an extensive review of the literature, Uchino [75] noted that individuals who perceive greater social support in their entourage are more likely to survive in the years following heart attack than those who perceive less social support, even when controlling for risk factors such as smoking and disease severity. Greenman, Tasse and Tulloch (in press) also note that the quality of the marital relationship has a strong link to heart health. It is therefore becoming increasingly apparent that effective treatments for PTSD in cardiac populations ought to involve patients’ significant others. The notion of including relationship partners or other significant figures in patients’ lives into their PTSD treatment makes sense when one considers that PTSD is primarily a disorder of affect regulation [76], and when people are able to create and maintain safe emotional connections to others they are better able to modulate and control their strong negative emotions than when they tend to do so alone [77]. Emotionally Focused Therapy (EFT; [78]), an empirically supported treatment for couple distress [79] may therefore be an ideal treatment option for patients with cardiac disease and symptoms of PTSD because its primary aim is to help relationship partners cultivate the kinds of close emotional bonds that are known to attenuate fear responses in the brain [80,81,82]. Preliminary evidence suggests that EFT might help reduce symptoms of PTSD in the general population and in couples in which one partner has a cardiac illness, as indicated in a pilot study of EFT and couples facing heart disease [80].

In summary, more clinical trials are needed to evaluate the effectiveness of PTSD treatments such as CBT and EFT applied to cardiac populations, and to determine if the adverse cardiac effects on overall physical health outcomes may be reduced or reversed if treated successfully. We do not know if effectively treating PTSD prevents further development of CVD or recurrence of events in those with established disease. This is an area that is ripe for further research.

## 7. Conclusions

Currently available research suggests that cardiac patients experience PTSD at much higher rates than the general population. It appears that both the chicken and the egg are involved in this relationship; that is, PTSD may contribute to the development of cardiac disease, and cardiac-related experiences may lead to trauma symptoms and PTSD. Either way, these patients are a high-risk group for recurrent cardiac morbidity and mortality via biological and/or behavioral mechanisms. Primary care and cardiac-specific clinicians need to routinely screen for trauma symptoms and, when elevated, refer to mental health treatment to prevent detrimental health outcomes. Greater integration of mental health services in these primary and specialty-care clinics will better serve these high-risk patients, particularly if they involve members of patients’ social support networks.

## Authors Contributions

All authors conceived the review paper idea. Heather Tulloch and Vanessa Tassé completed the literature search. Heather Tulloch and Paul S. Greenman wrote the paper. All authors reviewed the content and implications put forth, and commented on and approved of the final manuscript. 
